# Comparing tuberculosis in children aged under 5 versus 5 to 14 years old in a rural hospital in southern Ethiopia: an 18-year retrospective cross-sectional study

**DOI:** 10.1186/s12889-019-7206-2

**Published:** 2019-07-02

**Authors:** José M. Ramos, Mario Pérez-Butragueño, Abraham Tesfamariam, Francisco Reyes, Gebre Tiziano, Jacob Endirays, Seble Balcha, Tamasghen Elala, Dejene Biru, Belén Comeche, Miguel Górgolas

**Affiliations:** 1 Department of Medicine and Pediatrics, Gambo Rural General Hospital, Shashamane, Ethiopia; 2 Department of Internal Medicine, Alicante General University Hospital, ISABIAL, Calle Pintor Baeza, 12, 03010 Alicante, Spain; 3 Miguel Hernández University of Elche, Alicante, Spain; 4 Department of Pediatrics, Infanta Leonor University Hospital, Madrid, Spain; 5 National Referral Unit for Tropical Disease, Infectious Diseases Department, Ramón y Cajal University Hospital, IRICYS, Madrid, Spain; 6 Division of Infectious Diseases, Jiménez Diaz University Hospital Foundation, and Autonomic University of Madrid, Madrid, Spain

**Keywords:** Tuberculosis, Childhood tuberculosis, Children under five, Children, Pulmonary tuberculosis

## Abstract

**Background:**

There are few data available about childhood tuberculosis (TB) in rural hospitals in low-income countries. We assessed differences in epidemiological characteristics and treatment outcomes in children with TB aged 0–4 versus 5–14 years in rural Ethiopia.

**Methods:**

For this retrospective cross-sectional study, we analyzed childhood TB registers from a rural Ethiopian hospital. We collected data on the number of cases, type of TB, and treatment outcomes using standard definitions. By means of binary and logistic regression analyses, data were compared from 1998 to 2015 in children aged under 5 versus those aged 5–14 years.

**Results:**

We included 1282 TB patients: 583 (45.5%) were under 5 years old, and 699 (54.5%) were aged 5–14 years. More than half (67.2%, *n* = 862) had pulmonary TB (PTB), which was more common in younger children (82.5%, 481/583) than in older ones (54.5%, 381/699; *p* < 0.001). Most cases of PTB (87.5%, 754/862) were smear negative, including virtually all (99.6%, 479/481) younger children and most older ones (72.2%, *n* = 275/381; *p* < 0.001). The most common types of extrapulmonary TB (EPTB) were TB adenitis (54.5%, 229/420) and bone TB (20%, 84/420). Children under five showed a lower prevalence of adenitis TB (9.9% [58/583] versus 24.5% [171/699], *p* < 0.001), bone TB (2.9% [17/583] versus 9.6% [69/699], *p* < 0.001), and abdominal TB (0.9% [5/583] versus 6.3% [44/699], *p* < 0.001). Most diagnoses were new cases of TB (98.2%, 1259/1282). Overall, 63.5% (*n* = 814) of the children successfully completed treatment (< 5 years: 56.6%, 330/583; 5–14 years: 69.2%, 489/699; *p* < 0.001). In total, 16.3% (*n* = 209) transferred to another center (< 5 years: 19.4%, 113/583; 5–14 years: 13.7%, 96/699; *p* = 0.006). Thirteen percent of patients (*n* = 167) were lost to follow-up (< 5 years: 16.0%, 93/583; 5–14 years: 10.4%, 74/699; *p* = 0.004). Fifty-two (4.1%) children died (no age differences). Being aged 5–14 years was independently associated with successful treatment outcomes (adjusted odds ratio 1.59; 95% confidence interval: 1.16, 1.94, *p* = 0.002).

**Conclusions:**

We observed a very low diagnostic yield for spontaneous sputum smear in children with TB. In this rural setting in Ethiopia, very young children tend to present with new cases of smear-negative PTB. They have less EPTB than older children but more TB meningitis and show lower rates of treatment success.

**Electronic supplementary material:**

The online version of this article (10.1186/s12889-019-7206-2) contains supplementary material, which is available to authorized users.

## Background

Tuberculosis (TB) remains an important challenge for global health. The World Health Organization (WHO) estimated that in 2017 there were about 10 million incident cases of TB worldwide, and 10% of these were in children under 15 years old [1]. This is probably an underestimation, as childhood TB often remains undetected due to diagnostic challenges [2, 3]. The burden of this disease in children reflects its prevalence in adults [3].

Management of childhood TB poses challenges to low- and middle-income countries. Morbidity and mortality are high enough to make this one of the three primary infectious diseases related to poverty [4–6]. In 2017, about 233,000 children died from TB worldwide, which translates to 13% of total deaths in children [1].

A 2018 WHO report ranked Ethiopia 14th worldwide with regard to the global TB burden; 117,705 cases were reported in 2017, with an estimated incidence rate of 164 cases per 100,000 population per year. Of the reported cases in 2017, about 20,000 were in children under 15 years [1].

Given the natural history of TB, age remains the most important variable influencing the risk of disease progression following a primary infection with *Mycobacterium tuberculosis*. Disease manifestation patterns show clear associations with age at the time of primary infection. Immune-compromised children also carry an increased risk for TB [6], so it seems prudent to categorize all children under 2 years of age and immune-compromised children of any age as being at high-risk for this disease [7].

Although several studies have been published about childhood TB in Ethiopia, few have taken place in a rural environment. Moreover, they do not generally compare age groups in terms of epidemiology, disease manifestations, diagnosis or outcome [8–12], even though this information could be very useful for designing effective treatment strategies.

Ethiopia’s National Tuberculosis and Leprosy Control Programme (NTLCP), launched in 1992, helped standardize the registry of TB cases following WHO guidelines [13, 14]. This study describes the main characteristics of childhood TB in a rural hospital in Southern Ethiopia over 18 years, according to NTLCP registry data. We also compare epidemiological, clinical and outcome data in children aged under 5 versus those aged 5 to 14 years.

## Methods

### Study design, study site and period

This is a retrospective cross-sectional study from January 1998 to December 2015. We examined patient records in children aged less than 15 years old and diagnosed with TB at Gambo General Hospital, collecting data in line with the NTLCP reporting guidelines and WHO standards [13, 14].

Gambo General Hospital is a private rural general hospital with 135 beds. Since 2003, the hospital has had an inpatient feeding center with around 15–20 beds for treating severe acute malnutrition. It is 18 km from Arsi Negele, the capital of West Arsi Province; 45 km from Kuyera Hospital (the West Arsi reference hospital); and 250 km south of Addis Ababa. West Arsi Province has a population of 1.9 million, and Gambo General Hospital serves a population of about 95,000 people, living mostly in a rural setting and subsisting from agriculture and livestock.

In a study performed from July 2013 to June 2014 in the Hetosa District of Oromia Region, Ethiopia, the incidence of smear-positive pulmonary TB (PTB) was 214 (95% confidence interval [CI]: 163.3, 263.5)/ 100,000 persons per year [15]. A recent systematic review and meta-analysis of HIV infection in tuberculosis patients in Ethiopia from 2003 to 2017 showed that prevalence of HIV in the country was 23.4% (95% CI 19.6, 27.2). In the Oromia Region, where West Arsi is located, it was 20.9% (95% CI 17.8, 24.0) [16].

### Participants

Patients with a clinical suspicion of TB underwent a diagnostic study before starting treatment. If symptoms were consistent with PTB, patients provided three sputum samples, usually from spontaneous sputum, although gastric aspirates were taken in a few of the younger children. Two positive smears indicated smear-positive PTB. Patients with three negative smears received treatment with antibiotics, and if the symptoms continued, they were examined by chest X-ray. The following abnormalities on chest X-ray were suggestive of TB :(1) enlarged hilar lymph nodes and opacification in the lung tissue,(2) miliary mottling in lung tissue, and (3) cavitation (common with older children) or pleural or pericardial effusion. In case of respiratory symptoms, patients with suggestive chest X-rays whose condition did not improve with antibiotics were considered to have smear-negative PTB. Miliary tuberculosis was classified as pulmonary TB because of the lesions in the lungs [13, 14].

Extrapulmonary TB (EPTB) was generally diagnosed clinically. The investigations used to diagnose the common forms of EPTB were: (1) for peripheral lymph nodes, lymph node fine needle aspiration; (2) for TB meningitis, lumbar puncture; (3) for pleural effusion, chest X-ray plus pleural tap for biochemical analysis, cell count and culture; (4) for abdominal TB, abdominal ultrasound and ascites fluid analysis; (5) for osteoarticular TB, X-ray or joint tap, (6) for pericardial TB, ultrasound and pericardial tap. Tuberculous pleural effusion, without radiographic abnormalities in the lungs, was considered EPTB [13, 14]. Children with EPTB were screened for lung involvement by chest X-ray.

Patients with both PTB and EPTB were classified as having PTB; these cases were not included in both categories. The only exception was TB meningitis: if patients diagnosed with TB meningitis by lumbar puncture had an abnormal chest X-ray, they were considered as having TB meningitis. Bacteriological confirmation by culture was not undertaken. The Xpert MTB/RIF assay had not yet been implemented during the study period.

A TB diagnosis resulted in three potential management approaches: 1) hospital admission (seriously ill PTB or EPTB patients), 2) registration and ambulatory treatment at the TB clinic, or 3) transfer to the patient’s local TB clinic.

Clinicians completed case notification forms for all registered patients according to NTLCP reporting guidelines [13, 14], collecting data on: age, sex, weight, disease status (new case, previously treated TB, return after loss to follow-up, treatment failure), type of TB (smear-positive PTB, smear-negative PTB, EPTB), loss to follow-up, treatment failure, death or transfer, HIV status, and other clinico-epidemiological data. In this study, and in line with the NTLCP, patients were considered to have EPTB only in the absence of PTB. The registry does not include household contact data. Patients who were cured or completed treatment were categorized as having successful treatment, while unfavorable outcomes were defined as patients lost to follow-up, dying, those with treatment failure, and transfers.

The treatment scheme changed during the period of study, as shown in Additional file 1: Annex 1.

### Statistical analysis

All collected data were double-checked for completeness, entered into Excel, and exported to SPSS version 22.0 for analysis. We report variables of interest as frequencies or means. We performed the chi-square test and Fisher’s exact test to compare variables in univariate analysis, expressing results as crude odds ratios (ORc) with their 95% CIs. We then fit a logistic regression model to identify predictors of treatment outcome based on the adjusted odds ratio (ORa) and 95% CI. *P* values of less than 0.05 were considered statistically significant.

### Ethical approval

As the study was retrospective in nature, informed consent was not required from patients’ parents or legal guardians; however, all records were anonymized prior to analysis. The approved Local Research and Publication Committee of the Gambo General Hospital and the Health Unit and Ethical Review Committee of the Ethiopian Catholic Secretary approved this waiver as well as the protocol and the study (permit number: GH/MSMHF/710).

## Results

### General epidemiological data

**Fig. 1:**
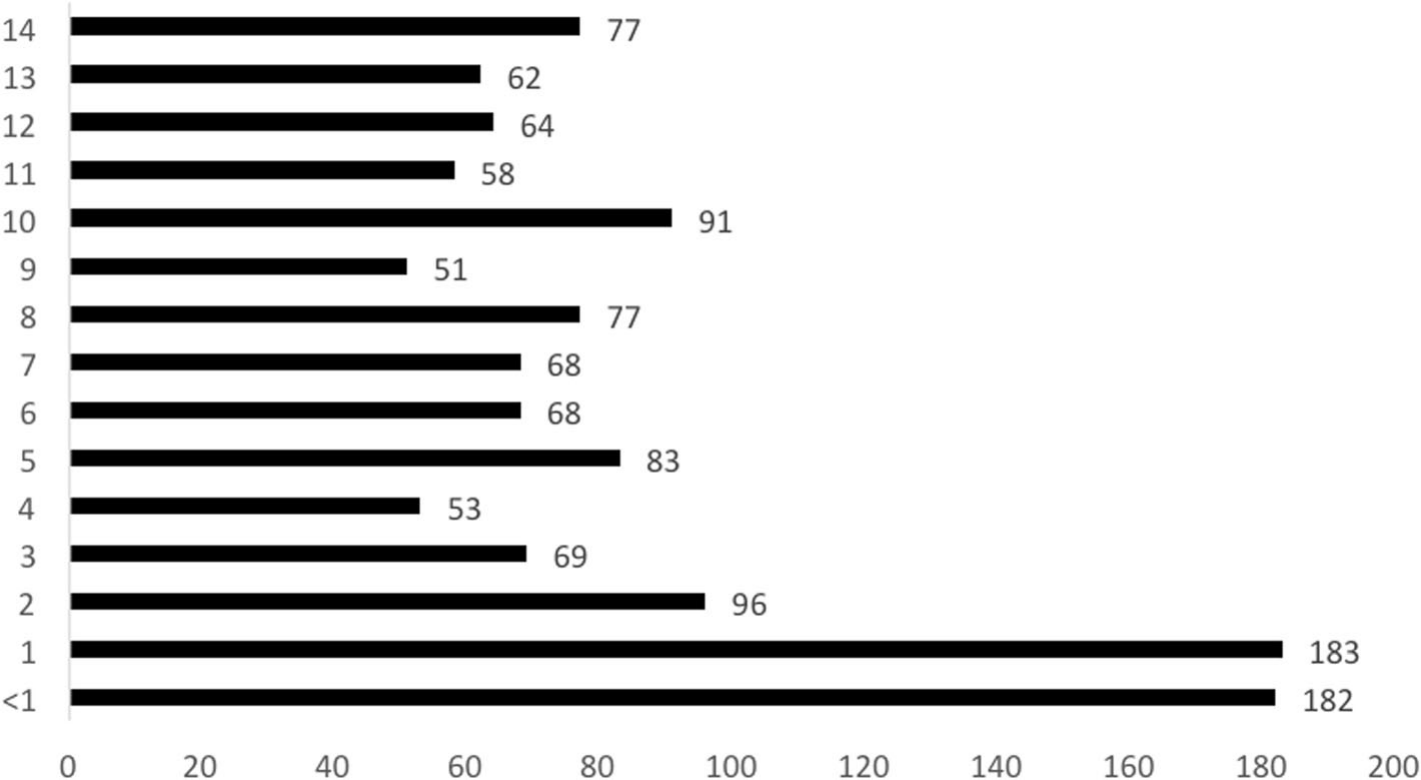
Number of children with tuberculosis, by age

**Fig. 2:**
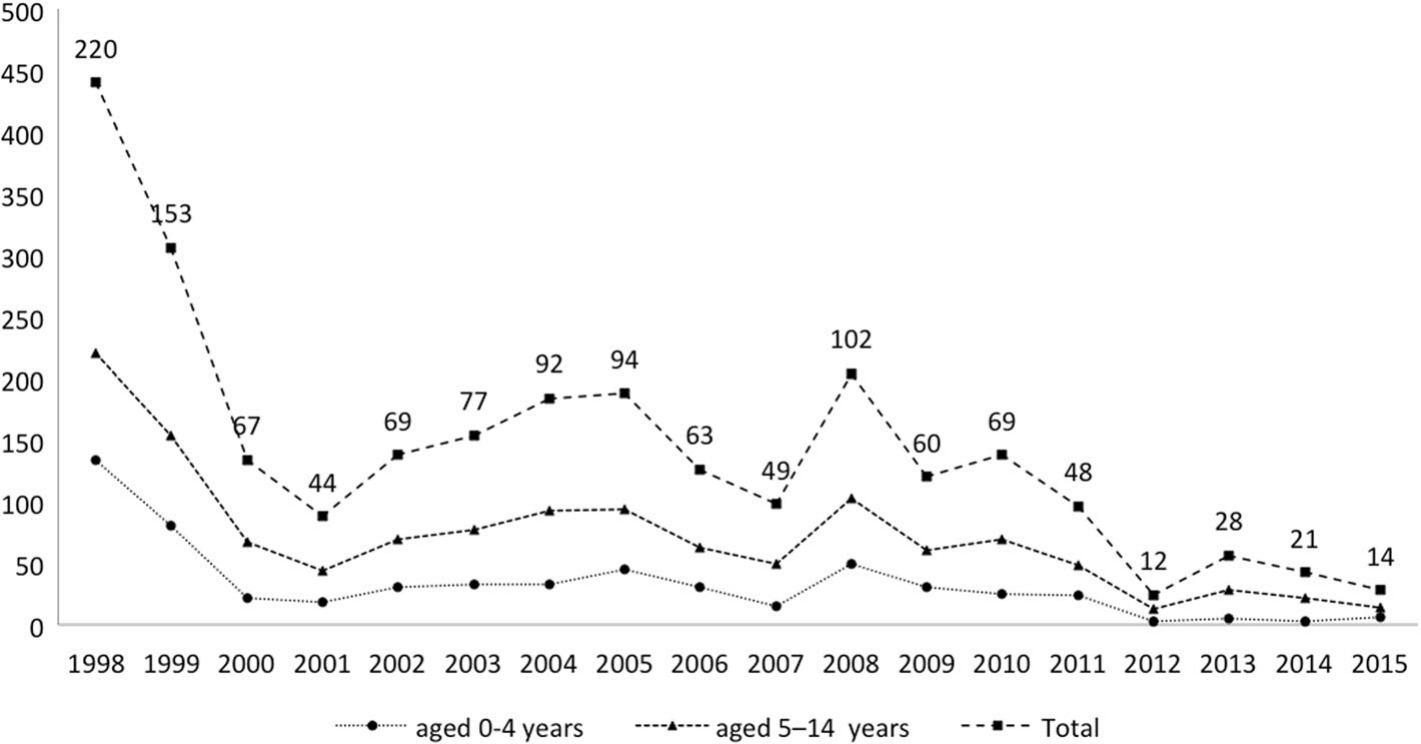
Tuberculosis cases in children total, aged 0–4 years, and 5–14 years, 1998 to 2015

From 1998 to 2015, 3534 patients were diagnosed with TB, including 1282 (36.3%) children: 583 of them (45.5%) were aged 0 to 4, and 699 (54.5%) were 5 to 14 years old. Figure 1 shows the distribution by age. Gender distribution did not differ according to age groups. The annual number of cases decreased over time, from more than 260 cases in the first 2 years to 20 cases at the end of the study (Fig. 2).

### Epidemiological and clinical characteristics of children under 5 versus children aged 5–14

Diagnostic results showed that PTB was the main type of TB (67.2%), and it was more common in younger children (82.5%) than in older ones (54.5%) (*p* < 0.001). Most (87.5%) cases of PTB were smear negative; this result was more common in children under five (99.6%) than in older ones (72.2%; *p* < 0.001) (Table 1).

**Table 1:** Epidemiological and clinical characteristics in children with tuberculosis: children aged 0–4 years versus 5–14 years

	Total (*N* = 1282) n (%)	0–4 years (*N* = 583) n (%)	5–14 years (*N* = 699) n (%)	Crude odds ratio (95% CI)	*P* value
Sex					
Boys	674 (52.6)	319 (54.7)	355 (50.8)	1	
Girls	608 (47.4)	264 (45.3)	344 (49.2)	0.85 (0.69, 1.06)	0.126
TB category					
Pulmonary TB	862 (67.2)	481 (82.5)	381 (54.5)	3.93 (3.03, 5.10)	< 0.001
Extrapulmonary TB	420 (32.8)	102 (17.5)	318 (45.8)	1	
Type of pulmonary TB (*N* = 862)					
Smear positive	108 (12.5)	2 (0.4)	106 (27.8)	1	
Smear negative	692 (80.3)	437 (90.8)	275 (66.9)	0.01 (0.00, 0.04)	< 0.001
Smear missing	62 (7.2)	42 (8.7)	20 (5.3)	–	
Type of extrapulmonary TB					
Adenitis TB	229 (17.9)	58 (9.9)	171 (24.5)	0.34 (0.24, 0.48)	< 0.001
Bone TB	84 (6.6)	17 (2.9)	67 (9.6)	0.28 (0.16, 0.48)	< 0.001
Abdominal TB	49 (3.8)	5 (0.9)	44 (6.3)	0.12 (0.05, 0.32)	< 0.001
TB meningitis	26 (2.0)	15 (2.6)	11 (1.6)	1.65 (0.75, 3.62)	0.205
Other and unclassified	32 (2.5)	7 (1.2)	25 (3.6)	0.32 (0.13, 0.75)	0.01
Category of TB					
New	1259 (98.2)	579 (99.3)	680 (97.3)	4.04 (1.37, 11.95)	0.006
Transfer	10 (0.6)	4 (0.7)	6 (0.9)	0.79 (0.22, 2.84)	0.763
Failure	8 (0.6)	0 (0.0)	8 (1.1)	NA	0.009
Relapse	5 (0.4)	0 (0.0)	5 (0.7)	NA	0.041
HIV status					
Known	400 (31.2)	157 (33.2)	237 (44.4)	1	
Unknown	882 (68.8)	426 (66.8)	457 (35.6)	0.69 (0.54, 0.87)	0.003
HIV status results (*N* = 400)					
Negative	391 (97.8)	154 (98.1)	237 (97.5)	1	
Positive	9 (2.3)	3 (1.9)	(2.5)	0.76 (1.19, 3.12)	0.999
Hospital admission					
No	772 (67.1)	303 (52.0)	469 (67.1)	1	
Yes	510 (39.8)	280 (48.0)	230 (32.9)	1.88 (1.50, 2.36)	< 0.001

*CI* confidence interval, *HIV* human immunodeficiency virus

TB adenitis was the most common type of EPTB (54.5%), followed by bone TB (20%), abdominal TB (11.7%) and TB meningitis (6.2%). Children under five showed a lower prevalence of adenitis TB (9.9% [58/583] versus 24.5% [171/699], *p* < 0.001), bone TB (2.9% [17/583] versus 9.6% [69/699], *p* < 0.001) and abdominal TB (0.9% [5/583] versus 6.3% [44/699], *p* < 0.001). TB meningitis was slightly more common in the younger age group (2.6% versus 1.6%).

Most patients were registered with new cases of TB, especially younger children (99.3% versus 97.3%; *p* < 0.006). Indeed, no TB cases in the under-five group showed failure or previous treatment, compared to eight and five cases, respectively, in older children (*p* = 0.009 and *p* = 0.041, respectively).

HIV status was tested in 31.2% of the children (33.2% of children under 5, and 44.4% of children aged 5–14; *p* = 0.003). Only 2.3% of the patients tested had a positive result. Determination of HIV status increased dramatically following implementation of the NTLCP in 2005 (5.2% before 2005, 99.4% afterwards).

Of 1282 total children, 510 (39.8%) were admitted, with a predominance of young children among those hospitalized (*p* < 0.001) (Table 1).

### Treatment outcome

**Fig. 3:**
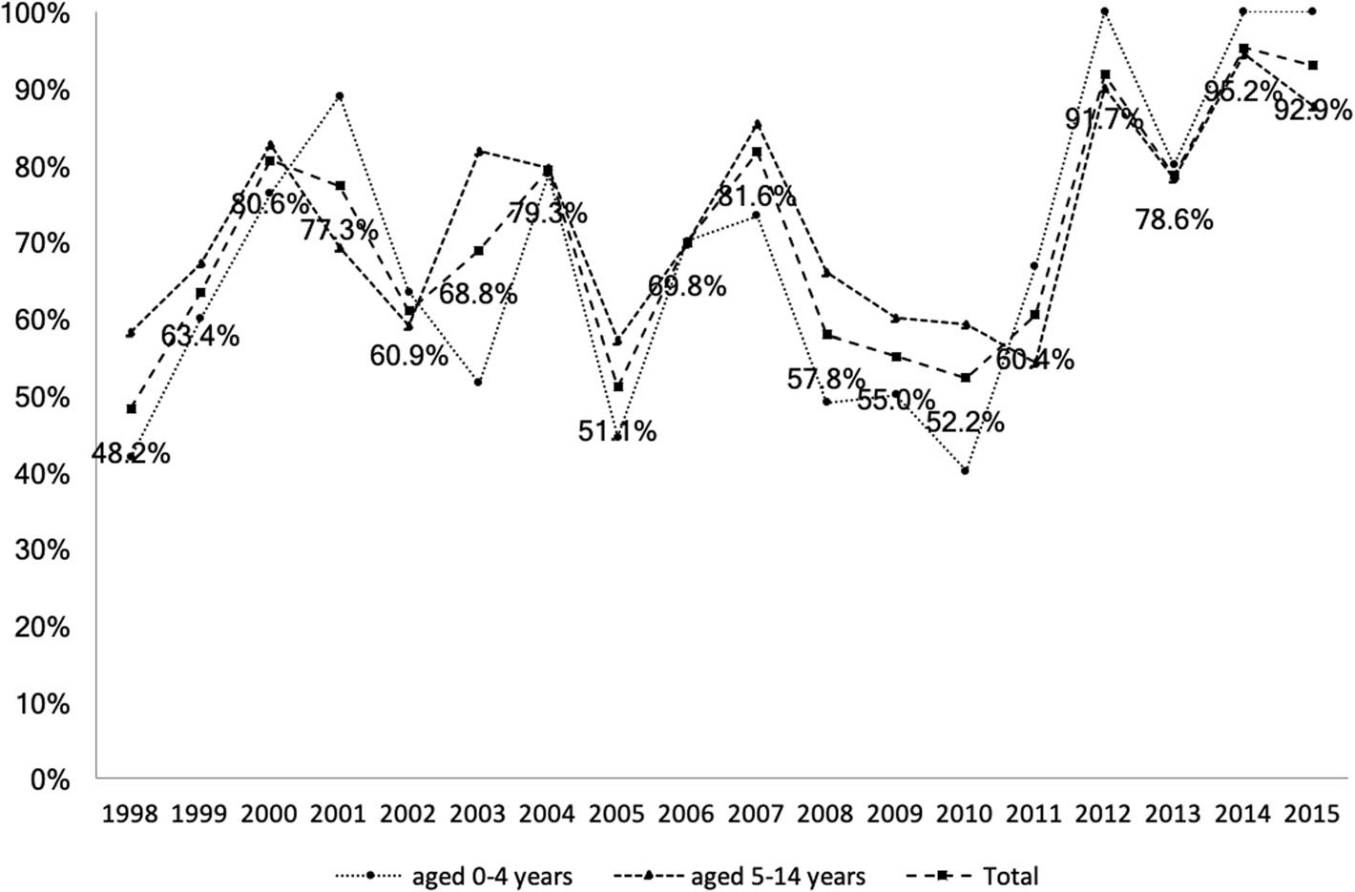
Percentage of treatment success rates in childhood tuberculosis, 1998 to 2015. Values of percentage of treatment success in total cases of childhood tuberculosis

Treatment outcomes were: 814 (63.5%) had successful treatment (56.6% in children < 5 years and 69.2% in children aged 5–14; *p* < 0.001); 209 (16.3%) transferred to another center (19.4% versus 13.7%; *p* = 0.006); 167 (13%) were lost to follow-up (16.0% versus 10.4%; *p* = 0.004); and 52 (4.1%) died (4.1% versus 4.0%; *p* = 0.999; Table 2). Figure 3 shows the successful treatment rates over time in childhood TB; these were quite variable.

**Table 2:** Treatment outcomes in children with tuberculosis: children aged 0–4 years versus 5–14 years

	Total (*N* = 1282) n (%)	0–4 years (*N* = 583) n (%)	5–14 years (*N* = 699) n (%)	Crude odds ratio (95% CI)	*P* value
Success	814 (63.5)	330 (56.6)	484 (69.2)	0.58 (0.46, 0.73)	< 0.001
Transfer	209 (16.3)	113 (19.4)	96 (13.7)	1.51 (1.12, 2.03)	0.006
Loss to follow-up	167 (13.0)	93 (16.0)	74 (10.6)	1.60 (1.15, 2.22)	0.004
Death	52 (4.1)	24 (4.1)	28 (4.0)	1.03 (0.59, 1.79)	0.999
Failure	3 (0.2)	1 (0.2)	2 (0.3)	0.60 (0.05, 6.62)	0.999
Missing data	37 (2.9)	22 (3.8)	15 (2.1)	–	–

*CI* confidence interval

Table 3 summarizes treatment outcome predictors in children with TB. In multivariable logistic regression, the only variable independently associated with successful treatment outcomes was being aged 5–14 years (ORa 1.59; 95% CI: 1.16, 1.94, *p* = 0.002).

**Table 3:** Predictors of treatment outcome of childhood tuberculosis (TB)

Characteristics	Treatment outcome		Univariate analysis		Multivariate analysis	
	Successful No (%)	Unfavorable No (%)	ORc (95% CI)	P value	ORa (95% CI)	*P* value
Sex						
Boys	418 (51.4)	256 (54.7)	Ref.		Ref.	
Girls	396 (48.6)	212 (45.3)	0.87 (0.69, 1.09)	0.248	0.89 (0.70, 1.12)	0.327
Age group						
Under 5	330 (40.5)	243 (54.1)	Ref.		Ref.	
5–14 years	485 (59.5)	215 (45.9)	1.72 (1.37, 2.17)	< 0.001	1.52 (1.16, 1.94)	0.002
Type of TB						
PTBSP	80 (9.8)	28 (6.0)	Ref.		Ref.	
PTBSN	445 (54.7)	309 (66.0)	1.98 (1.26, 3.12)	0.003	1.51 (0.93, 2.47)	0.092
EPTB	289 (35.5)	131 (28.0)	1.29 (0.80, 2.08)	0.288	1.15 (0.71, 1.88)	0.553
New TB						
No	16 (2.0)	7 (1.5)	Ref.		Ref.	
Yes	798 (98.0)	461 (98.5)	0.75 (0.30, 1,95)	0.542	0.98 (0.39, 2.54)	0.990
HIV status						
Negative	249 (30.6)	142 (30.3)	Ref.		Ref.	
Positive	7 (0.9)	2 (0.4)	1.01 (0.79, 1.30)	0.887	0.98 (0.76, 1.27)	0.926
Unknown	558 (68.6)	324 (69.2)	0.50 (0.10, 2.44)	0.393	0.50 (0.10, 2.48)	0.386

*PTBSP* pulmonary tuberculosis smear positive, *PTBSN* pulmonary tuberculosis smear-negative, *EPTB* extrapulmonary tuberculosis, *HIV* human immunodeficiency virus, *ORc* crude odds ratio, *ORa* adjusted odds ratio

## Discussion

More than a third (36.3%) of the total recorded cases of TB in our study population occurred in children—a higher proportion than reported elsewhere in Ethiopia [9, 11, 17], in the national estimate (16.1%) [18], and in other areas of sub-Saharan Africa [19]. Several factors may have contributed to these results. As our study took place in a private hospital, it offered several services, such as X-ray diagnosis and cheaper treatment, that may not have been present in public facilities. At the same time, the hospital has a referral malnutrition clinic, where children with malnutrition are admitted for a minimum fee, and these children may have TB. Some overdiagnosis of child TB cases may have also been possible.

Given the scant data available on childhood TB (and the dominance of high-income settings where studies exist), in 2007 WHO called for additional research to characterize the global epidemiology of the disease [20].

Nearly half (45.5%) of the total childhood TB cases were in children under five, which is consistent with results from a previous study in Tanzania [21] but higher than in other studies in Ethiopia and other low- and middle-income countries, where the proportion of TB cases in this group is estimated at 11 to 23% [8, 9, 22]. Overall, the heterogeneous estimates reflect limitations in available data both at a country and regional level.

Childhood TB is mostly due to recent transmission, so its burden provides a good proxy measure for TB prevalence in the community [19]. Therefore, the high rates of childhood TB that we observed suggest that the community bears a high burden of untreated disease. Moreover, 41.6% of Ethiopia’s population is aged under 15 years [23], and this is also relevant to the burden.

There has been a decrease in prevalence of tuberculosis in Ethiopia over the past 15 years [1]. In our study, the annual case count also decreased over the study period, especially in the final 4 years, as the number of patients transferred to local health centers (and whose cases were therefore registered there instead of in the hospital) increased as these centers were newly constructed in the area. We consider that most of the reduction in numbers is due to a shift in patients receiving treatment in new community centers rather than in the hospital.

Most cases (98.2%) were new TB rather than previously treated TB. This proportion was slightly higher than in other studies of childhood TB (92%) [8, 9]. The younger age group presented with more new cases, which is logical, as for the most part such children are too young to have already been treated for the disease. In general, previously treated TB and transfer cases in our study were infrequent.

PTB was the most common type of TB in our children, which is consistent with the literature [18]. In our study, microbiological diagnosis (only acid-fast bacillus smear positivity, as bacteriological confirmation by culture was not done) was very low, as in other studies in Africa [21]. Just 12.5% of the PTB cases were smear positive, which is also in line with previous reports in Ethiopia [9, 11]. Most of the patients with smear-positive PTB were aged 5–14 years. Children develop mainly primary PTB presenting with lymph nodes and areas of parenchymal infiltration, and they tend to have low bacillary load and immunological changes, which explains this result [9]. Moreover, young children rarely produce sputum samples for smear microscopy, so they are generally diagnosed based on clinical examination and chest X-ray [24]. This was also true in our study, in line with NTLCP/WHO guidelines on diagnosing children [9]. The probability of smear positive sputum is positively correlated with the child’s age [8, 9, 24, 25].

Using other sample types, such as gastric aspirate, results in a higher diagnostic yield than spontaneous sputum in children. The Xpert MTB/RIF assay in sputum and gastric aspirates may identify *M. tuberculosis* in a greater percentage of cases in this group [25–27]. Thus, the gradual introduction of Xpert MTB/RIF in gastric aspirate and induced sputum samples could help to improve diagnosis in children under 15 in rural settings [26, 27].

EPTB made up a third of the childhood TB cases, which is a lower proportion than in the studies by Hailu et al. [9] and Tilahun and Gebre-Selassie [8], where EPTB accounted for about half. There were more cases of EPTB in the older age group compared to the younger children (45.8% versus 17.5%), which is consistent with Hailu et al’s [9] results in Ethiopia and Mtabho et al’s [22] observations in Tanzania. In these studies, as in ours, a patient with both PTB and EPTB was classified as having PTB.

On the other hand, there was a slightly higher proportion of TB meningitis in children under five (2.6% versus 1.6% of total TB); it is well known that this type of TB is more frequent in children under three [27].

Abdominal TB appeared less frequently in younger children (4.9%) compared to older ones (13.8%). Although this variant is rare in children, most reported cases are in low- and middle-income countries with a considerable TB burden. Consistent with our results, most cases of abdominal TB are in patients aged over five [28, 29]. Diagnosis is extremely challenging; our patients were diagnosed by abdominal ultrasound, ascitic fluid analysis (lymphocytosis) and clinical suggestion because microbiological confirmation was not possible. Only a few cases had histopathological diagnosis.

Overall TB/HIV co-infection in our series showed a prevalence of 2.3%, much lower than elsewhere in Ethiopia, where it ranges from 10 to 28.2% [8, 9, 30–34]; in other African countries, where it ranges from 14 to 50% [19, 31, 33]; and in Southeast Asia [22, 35]. The low proportion of our patients tested for HIV precludes any conclusion on why the prevalence was so low; however, we can cautiously speculate that it is because HIV is less prevalent in rural populations than in other areas of Ethiopia [36, 37].

In our study, 39.8% of the children were admitted to hospital. Some patients were hospitalized for reasons other than TB, such as severe acute malnutrition, and were only then diagnosed with TB. Hospitalization was more frequent in the under-five group.

TB programs in sub-Saharan Africa rarely assess treatment outcomes in children [18]. The WHO considers an overall treatment success rate of 85% to indicate good quality TB case management [38]. Our success rate—63.5%—was low compared to that achieved elsewhere in Ethiopia (~ 85%) [8, 9, 11] and Africa [22]. If we assume that the patients transferred to other TB clinics (16.3%) fully completed the course of treatment, though, the success rate of 80% is closer to the WHO target [38].

Indeed, the transfer rate was relatively high in our series [8, 9, 11] because patients beginning treatment during hospitalization in Gambo General are registered in their local health center upon discharge, and they complete therapy there [39]. Due to limitations in these registry data, we could not determine the final treatment outcome in these patients [39].

In our series, the proportion of patients who successfully completed treatment was lower in children under 5 than in those aged 5–14 (56.6% versus 69.2%), but the transfer rate was higher (19.4% versus 13.7%). The rate of treatment loss to follow-up was 13%—higher than in other studies in Ethiopia with more urban and periurban settings [8, 9] and in Tanzania [21]. However, our loss to follow-up rate was marginally lower than the 15% reported by Adejumo et al. [19] in Nigeria.

With regard to the mortality rate, the 4.1% we observed is within the range reported in other studies of childhood TB in Ethiopia [8, 9, 11, 33]. Elsewhere in Africa, mortality has higher: 10.5% in Botswana [40], 10.6% in Tanzania [21] and 17% in Malawi [41].

Young children, particularly those under two years of age, are at higher risk of dying from TB because their immune system is still immature [9, 17, 27, 42]. We did not see a difference in fatal outcomes in our series based on age, although this result may be confounded by the lack of treatment outcome data for transfer patients.

In terms of treatment outcome predictors, age group was the only variable that was independently associated with treatment success. This outcome was significantly higher in 5–14-year-olds compared to the under-5 group. Other studies have reported similar findings [8, 9, 18, 43].

In contrast, other series have reported significant associations between treatment failure and HIV co-infection, unknown serostatus and malnutrition [2, 9, 39, 44].

The findings of this study must be interpreted in light of its limitations. First, it is common to find both PTB and EPTB in the same pediatric patient, but in this study the variable was dichotomized (EPTB only in the absence of PTB), following WHO definitions. In children, this classification can obscure very important information about serious cases of TB, for instance if the child is diagnosed as having PTB but also has TB meningitis, which could be much more life threatening. Secondly, we were unable to collect sociological data and only limited data about HIV infection, which might have affected the risk of a poor outcome. Finally, Gambo General Hospital is a private hospital, and patients were often referred from other health facilities for diagnosis and admission but then transferred to their local health center following the intensive treatment phase. Thus, we might have underestimated treatment failure and mortality rates. Additional limitations are the retrospective and hospital-based nature of the study, which could cause bias. Moreover, there also seem to be recent changes in health system in the area, which could depress estimates of prevalence.

## Conclusions

Data collection on childhood TB in rural health facilities is important for understanding the epidemiology of this disease. Because spontaneous sputum smear has a very low diagnostic yield in children in low- and middle-income countries (and particularly in children under five), alternative diagnostic methods, such as Xpert MTB/RIF in gastric aspirates, become more important. In this rural setting in Ethiopia, children under five usually present with new smear-negative PTB. They have less EPTB than children aged 5 to 14 but more TB meningitis. Younger children are admitted more frequently and show lower rates of successful treatment.

## Additional file

Additional file 1: Annex 1. Treatment regimen according to type of tuberculosis following the Tuberculosis and Leprosy Prevention and Control Programme in Ethiopia. (DOCX 88 kb)

## Data Availability

Since data were retrospectively collected from a hospital database and were not publicly available, we sought and received permission to use them from Publication Committee of the Gambo General Hospital. The datasets generated and/or analyzed during the performance of the study are available from the corresponding author upon request.

## Abbreviations

EPTB: Extrapulmonary tuberculosis
HIV: Human immunodeficiency virus
ORa: Adjusted odds ratio
ORc: Crude odds ratio
PTB: Pulmonary tuberculosis
TB: Tuberculosis
WHO: World Health Organization
