# Supplementary material for: Comparing tuberculosis in children aged under 5 versus 5 to 14 years old in a rural hospital in southern Ethiopia: an 18-year retrospective cross-sectional study
Source: BMC Public Health. 2019 Jul 2;19:856. doi: 10.1186/s12889-019-7206-2 (PMC6604275; doi:10.1186/s12889-019-7206-2)
Supplement: Supplementary file 1 — Annex 1. Treatment regimen according to type of tuberculosis following the Tuberculosis and Leprosy Prevention and Control Programme in Ethiopia. (DOCX 88 kb) [file 12889_2019_7206_MOESM1_ESM.docx]

Annex

Annex 1. Scheme of treatment according to type of tuberculosis following the Tuberculosis and Leprosy Prevention and Control Program in Ethiopia

|  | 2013 Guidelines | 2008 Guidelines | 2005 Guidelines | 2002 Guidelines | 1999 Guidelines |
| --- | --- | --- | --- | --- | --- |
| Category I:  Short course chemotherapy for smear-positive PTB and seriously ill smear-negative PTB and EPTB cases | 2 (ERHZ)/4(RH)* | 2 (ERHZ)/4(RH)** | Children < 7 years  2 (SRHZ)/4(RH)  Children > 6 years  2 (ERHZ)/4(RH) | Children < 7 years  2 (SRHZ)/4(RH)  Children > 6 years  2 (SRHZ)/6(EH) | Children  2(SRHZ)/6(TH) |
| Category II:  Retreatment regimen | 3ERHZ/5 (ERH) | 2SERHZ/1ERHZ/5 (ERH)3 | 2SERHZ/1ERHZ/5 (ERH)3 |  |  |
| Category IIIa:  Short course chemotherapy for smear negative PTB, EPTB who are not seriously ill | *** | 2 (RHZ) / 4 (RH) | Children < 7 year  2 (RHZ) / 4 (RH)  Children >6 year  2 (RHZ)/ 6 (EH) or  2 (RHZ) / 4 (RH) | Children < 7 year  2 (RHZ) / 4 (RH)  Children >6 year  2 (RHZ)/ 6 (EH) |  |
| Category IIIb: Long course chemotherapy for smear negative PTB, EPTB who are not seriously ill |  |  |  |  | Adults and Children  2 (STH)/10 (TH) |

PTB: pulmonary tuberculosis; EPTB: extrapulmonary tuberculosis; H, isoniazid; R, rifampicin; S: streptomycin; Z: pirazinamide; E: Etambutol

* Children with suspected or confirmed tuberculous meningitis and osteo-articular TB should be treated with a four-drug regimen (HRZE) for 2 months, followed by a two-drug regimen (HR) for 10 months, the total duration of treatment being 12 months.

** TB Meningitis (Guidelines 2008): 2(ERHS)/4(RH) [streptomycin replaces etambutol]

*** This category disappears in guideline of 2013.
